# Pulmonary Sarcoidosis and Immune Dysregulation: A Pilot Study on Possible Correlation

**DOI:** 10.3390/diagnostics13182899

**Published:** 2023-09-11

**Authors:** Rossella Cifaldi, Francesco Salton, Paola Confalonieri, Liliana Trotta, Mariangela Barbieri, Luca Ruggero, Gianmaria Valeri, Riccardo Pozzan, Rossana Della Porta, Metka Kodric, Elisa Baratella, Mattia Bellan, Selene Lerda, Michael Hughes, Marco Confalonieri, Maria Assunta Cova, Ilaria Gandin, Lucrezia Mondini, Barbara Ruaro

**Affiliations:** 1 Pulmonology Unit, Department of Medical Surgical and Healt Sciencies, Hospital of Cattinara, University of Trieste, 34149 Trieste, Italy; 2 Department of Radiology, Hospital of Cattinara, University of Trieste, 34149 Trieste, Italy; 3 Department of Translational Medicine, Università del Piemonte Orientale (UPO), 28100 Novara, Italy; 4 Center for Autoimmune and Allergic Disease (CAAD), Università del Piemonte Orientale (UPO), 28100 Novara, Italy; 5 Department of Internal Medicine, University Hospital, Maggiore della Carità, 28100 Novara, Italy; 6 Management Specialization School, University of Milan, 20149 Milano, Italy; 7 Division of Musculoskeletal and Dermatological Sciences, Faculty of Biology, Medicine and Health, The University of Manchester & Salford Royal NHS Foundation Trust, Manchester M6 8HD, UK; 8 Biostatistics Unit, Department of Medical Sciences, University of Trieste, 34149 Trieste, Italy

**Keywords:** sarcoidosis, lymphopenia, severity index, lymphocyte typing, immune dysregulation

## Abstract

Background: Sarcoidosis is a systemic inflammatory disease characterized by an altered inflammatory response. Objective: The aim of this study was to evaluate whether immune system alterations detected by lymphocyte typing in peripheral blood correlate with the severity of sarcoidosis, calculated according to two separate severity scores proposed by Wasfi in 2006 and Hamzeh in 2010. Materials and Methods: Eighty-one patients were recruited, and clinical data and laboratory tests at the time of diagnosis were obtained in order to assess the severity index score and investigate any statistically significant correlation with the cytofluorimetry data. Results: Our data demonstrated that none of the two scores show an association with the level of total lymphocytes or lymphocyte subclasses. Limitations: First of all, the sample taken into consideration is small. The assessment was performed only at disease onset and not during the disease. Furthermore, the severity scores do not take into account disease activity (measured by PET/CT or gallium scintigraphy). Conclusions: Lymphocyte subpopulation values at the time of diagnosis do not appear to correlate with disease severity at onset.

## 1. Introduction

Sarcoidosis is a systemic granulomatous inflammatory disease that primarily affects the lungs and mediastinal lymph nodes, with intrathoracic involvement in 90% of cases. However, it can potentially involve any organ or system [1,2,3]. The prevalence of this disease varies widely among countries around the world, being highest in Sweden (140–160 per 100,000 people) and in African Americans [1]. Sarcoidosis is a frequently insidious disease, and diagnosis is often a chance finding at a routine chest radiography carried out for other reasons [1,2,3]. The chest radiograph typically evidences bilateral hilar adenopathy [1,2,3,4]. Lung involvement is common, and symptoms may include cough, dyspnea, and chest pain. Extrapulmonary symptoms may involve the joints, skin, microcirculation, and eyes [2,3,4,5,6,7,8,9]. The increased angiotensin-converting enzyme levels are commonly observed, although this finding is not diagnostic, and histopathologic confirmation is required to confirm the diagnosis [1,2,3,4,5,6]. This biopsy is usually obtained from the most peripheral site possible [3,4,5,6,7]. The main typical lesion of sarcoidosis is the noncaseous granuloma, a well-formed, non-necrotizing, epithelioid cell interstitial granuloma associated with hyaline fibrosis and characterized by a lymphangitic distribution, which can be found in the pleura, lung tissue, skin, cardiac or nervous system, or any other affected organ [2,4,5]. The central nucleus shows a predominant epithelioid cell, believed to be a differentiated mononuclear phagocyte. CD4+ T lymphocytes and mature macrophages are concentrated in the epithelioid core, while CD4+ and CD8+ T lymphocytes are visible at the periphery of the granuloma. Multinucleated giant cells are scattered throughout the inflammatory zone [6,7,8].

Dysregulation of the T cell-mediated immune response has also been observed in patients with sarcoidosis, associated with a decrease in antibody title. The immune response in the pathogenesis of sarcoidosis is characterized by a bias toward the Th1 response, with the release of cytokines and chemokines associated with this type of immune response (e.g., IFN-γ, TNF-a, and often IL-2). In contrast, low or undetectable levels of IL-4, IL-5, and Th2-associated chemokines and chemokine receptors are found in the sarcoidotic lung [6,7,8,9,10,11,12]. CD4+ regulatory T cells show reduced numbers and function in sarcoidosis, confirming that Treg lymphocytes play a role in the altered immune response of this disease [6,7,8,9,10,11,12].

In fact, Drake’s group, in 2013, showed that sarcoidosis results in decreased production of IL-2 and INFγ, resulting in reduced CD4+ T lymphocyte activity [6,7,8,9]. Another study conducted on CD17+ T cells showed an increase in patients with sarcoidosis compared with healthy subjects in both peripheral blood and BAL. However, it was later found that these cells produce much less INFγ than in healthy subjects, which could be essential for understanding the lack of action of macrophages and effector cells that then lead to granuloma formation [6,7,8,9]. Reduced production of INFγ and TNFa has also been found in iNKT lymphocytes; these abnormalities are indicative of a “depletion” of immune cell function, both of which correlate with a disease stage or circulating levels of iNKT [6]. In addition, in patients with severe sarcoidosis, there is a deficiency of B cells, particularly memory B cells, and an increase in naïve B cells and compensatory plasmablasts. In addition, a reduction in Nf-kB/p65, which is considered the key transcription factor for cytokine production after TCR and antigen-MHC binding, has been demonstrated [10,11]. In addition, the level of Nf-kB/p65 was also analyzed in CD4+ T cells, and it was seen that even in CD4+ naïve patients with severe sarcoidosis, the levels of this protein are very low. This indicates that in these patients, both T and B lymphocytes have an altered response to TCR-antigen-MHC stimulation and, thus, a reduction in IL-2 and INFγ production [13]. Several studies have uncovered aspects of this pathology that place it among autoimmune spectrum disorders by deepening and studying in detail the mechanisms so far known concerning the pathogenesis of sarcoidosis [14]. In particular, sarcoidosis appears to be associated with specific HLA antigens; several studies have also shown associations between sarcoidosis and other autoimmune diseases (rheumatoid arthritis, Sjogren’s syndrome, and Crohn’s disease), suggesting a possible common pathogenesis [14,15,16,17]. In particular, in support of these hypotheses, an interesting Italian study by Cattelan et al. examined the microcirculation of patients with sarcoidosis by nailfold capillaroscopy, finding nonspecific alterations compared to a healthy control population [16]. This important work, therefore, suggests an involvement of the microcirculation by sarcoidosis, linking this disease to other rheumatic diseases. It is also interesting to point out that the patients in this study were also subjected to a search for autoantibodies in the blood, showing that 42.3% of patients with sarcoidosis were positive for ANA [15,16]. This finding is even more interesting, considering that patients with other rheumatic diseases in their history were carefully excluded from the subgroup of sarcoidosis patients [16].

The purpose of our study is to analyze in our cohort of patients the existence of a correlation between the severity of sarcoid pathology at the time of diagnosis—according to the scores proposed in the literature—and the values of CD4+, CD8+, and CD19+ lymphocytes on peripheral blood at the same time, to understand whether these values can be used for an initial assessment of pathology.

## 2. Materials and Methods

### 2.1. Patient Population

After having given written informed consent, a total of 81 patients affected by sarcoidosis were enrolled from January 2019 to December 2022. The diagnosis was made during a routine clinical assessment in our pulmonology department, a referral center for sarcoidosis [4,5,18,19,20,21,22,23,24,25,26]. A complete medical history was collected and all patients were examined clinically. Demographic and clinical data (i.e., age, gender, smoking status, involvement of various organs and systems, and treatment regimens) were also recorded. For each patient, we searched for initial symptoms, divided into nonspecific and organ-specific involvement, as listed (see Table 1). 

**Figure 1:**
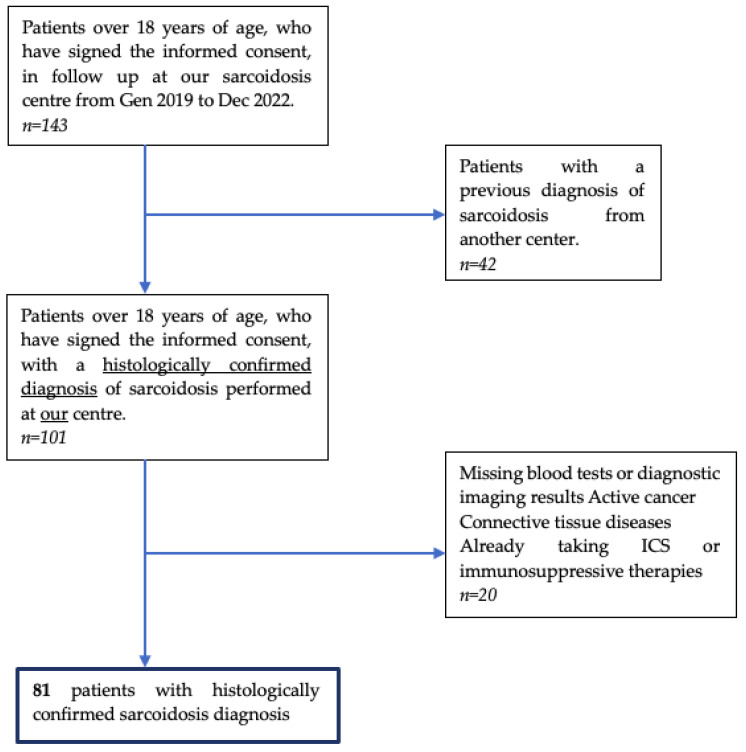
PRISMA diagram illustrating the patient selection process in our study (Gen: January; Dec: December; ICS Inhaled corticosteroids).

We excluded subjects with insufficient clinical information (i.e., age at diagnosis and smoking habit), missing blood tests or diagnostic imaging results, active cancer and connective tissue diseases, or patients who, for other reasons, were already receiving steroid therapy or another type of specific immunomodulatory therapy at the time of diagnosis. We excluded patients who came to our center with an already-known diagnosis of sarcoidosis. Furthermore, the patients we have included can be defined as ‘naïve’, i.e., without an established diagnosis. Inclusion criteria were also having been diagnosed with sarcoidosis [7] at our center, being 18 years or older, and volunteering to participate. The patient selection process is illustrated in the PRISMA diagram in Figure 1.

We recruited 81 patients, including 46 (57%) males and 35 (43%) females, with a mean age of 57 years (SD 12 years). All patients are of Caucasian race, except for 1 Afro-American patient. In total, 34 out of 81 (42%) patients are smokers. In the cohort, 74 (91%) patients at onset have pulmonary symptoms, 23 (28%) skin symptoms, 20 (25%) joint and muscle symptoms, and 16 (20%) cardiac symptoms. According to the Scadding score, 2 (2.5%) patients are classified into stage 0, 8 (9.9%) into stage 1, 23 (34%) into stage 2, 15 (19%) into stage 3, and 17 (21%) into stage 4. Regarding pulmonary function tests, 68 (84%) patients had an FEV1 between 70 and 100% predicted, 11 (14%) an FEV1 between 50 and 70% predicted, and 2 (2.5%) less than 50% predicted. Seventy-nine of the patients (98%) were initially given steroid therapy. Subsequently, nonsteroidal immunosuppressive therapy was introduced to 58 of them (73%). The characteristics of the patients are reported in Table 2. Blood tests (detailed in Section 2.2) and pulmonary function tests (detailed in Section 2.3) were performed for all patients at the time of diagnosis. 

### 2.2. Blood Tests

After obtaining written informed consent, a complete blood chemistry evaluation was made, i.e., a total blood count, peripheral blood values of CD4+, CD8+, CD19, the CD4/CD8 lymphocyte ratio, and ACE (angiotensin-converting enzyme) were collected for each patient at the time of diagnosis.

### 2.3. Pulmonary Function Tests

Pulmonary function tests (PFTs) are essential, readily available, and noninvasive [18,19]. The PFTs were performed in the pulmonology units of the University Hospital of Trieste, and the same operator and equipment evaluated all patients. Global spirometry values were recorded, i.e., functional vital capacity (FVC), forced expiratory volume in 1 s (FEV1), forced expiratory volume in 1 s/functional vital capacity (FEV1/FVC) ratio, also known as the Tiffeneau–Pinelli Index (IT), and diffusion capacity of carbon monoxide (DLCO). The employment of respiratory function tests in the management of patients with sarcoidosis, to date, is particularly useful for monitoring the long-term course of the disease [20,21,22,23,24].

### 2.4. Scores

Currently, there are no guidelines in the literature that have validated scores for determining the severity of sarcoidosis, so the characteristics of these patients have been used to calculate severity scores according to two separate formulas proposed by Wasfi et al. in 2006 [24] and Hamzeh et al. in 2010 [25]. 

Wasfi et al. [24] proposed a severity score consisting of Equation (1):

(1) $$Severityscore=11.47+3.51\left(C\right)+2.27\left(N\right)+1.41\left(IS\right)-0.033\left(Dlco\%\right)-0.047\left(\frac{FEV1}{FVC}\right)+1.23\left(AA\right)-0.027\left(FVC\%predicted\right)+0.52\left(Skin\right)$$

where *C* = 1 if there is cardiac involvement, 0 if not; *N* = 1 if there is neurologic involvement, 0 if not; *IS* = 1 if receiving noncorticosteroid immunosuppression therapy, 0 if not; *AA* = 1 if the subject was African American, 0 if not; and skin = 1 if there is skin involvement, 0 if not. The lower the score, the greater the severity of the disease. The second severity score, proposed by Hamzeh et al. [25], correlates the Scadding score with pulmonary function tests (FVC, Tiffeneau index, and DLCO), providing a sum ranging from 0 to 7, identifying increasing severity of the disease (resumed in Table 3) [25].

### 2.5. Statistical Analysis

Descriptive statistics are reported as means with standard deviation (SD), medians with interquartile ranges (IQR), or counts with percentages, as appropriate. Cross-sectional associations between the two severity scores and the blood values of CD4+, CD8+, CD19+, and total lymphocytes were investigated. Given the asymmetry in the distribution of the variables of interest, relationships were estimated with Spearman’s rank correlation coefficient. The statistical significance level was set at *p* < 0.05 or less according to Bonferroni correction, and *p* < 0.05 was considered indicative of suggestive evidence. All analyses were performed using the statistical software R version 4.1.2.

### 2.6. Ethics

The study was conducted in accordance with the principles of the Helsinki Declaration of 1975/83 and Good Clinical Practice guidelines and was approved by the Ethics Committee (CEUR number 3673, date of approval 2 March 2021). All patients signed the informed consent for the management of their clinical data and all analyses, as per University Hospital rules.

## 3. Results

### 3.1. Flow Cytometry Results

**Figure 2:**
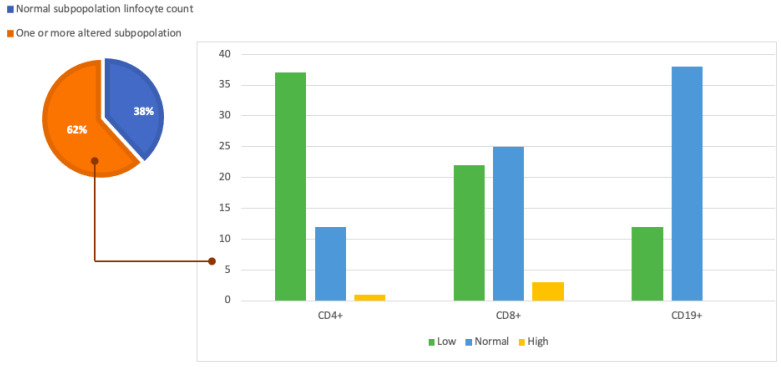
Graphic representation of our study population and cytofluorometry results. The pie chart on the left shows the proportion of patients in the population under analysis with normal lymphocyte counts (38%) and the proportion of the population with one or more subpopulations with altered lymphocyte counts (62%). The histogram on the right analyzes this latter part of the population in more detail and shows the number of patients with altered lymphocyte subpopulations divided by CD4+, CD8+, and CD19+.

Analyzing the values at flow cytometry showed that 21 patients (26%) had lymphopenia with total lymphocyte counts <1000 cells/µL. Twelve of these patients (57%) had low CD4+ (<500 cel/µL), CD8+ (<350 cells/µL), and CD19+ (<140 cel/µL) lymphocyte counts. Furthermore, 38 patients (47%) showed low levels of CD4+, 22 patients (27%) had low blood levels of CD8+, and 19 patients (23%) had low CD19+. More data about cytometry analysis are shown in Table 4 and Figure 2.

### 3.2. Correlation between the Two Severity Scores

**Figure 3:**
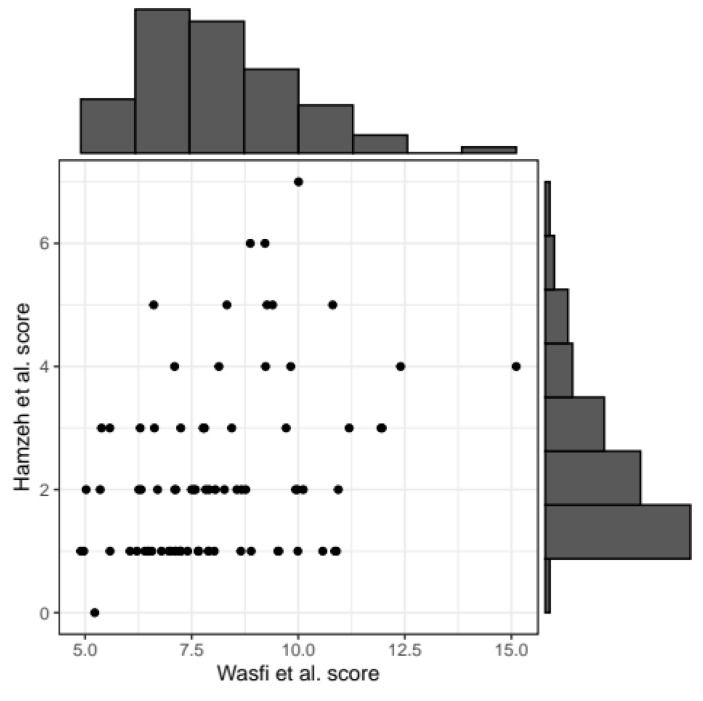
Scatterplot representing the correlation between the two scores. Black points correspond to patients’ values for the two scores. Histograms summarizing the distribution of Wasfi et al. [24] score (x-axis of scatterplot) and Hamzeh et al. [25] score (y-axis) are reported on the horizontal and vertical axis, respectively.

We first calculated the correlation between the Wasfi et al. [24] score and the Hamzeh et al. [25] score. The correlation coefficient between the two severity scores was estimated at r = 0.34 (*p*-value = 0.002). A graphical representation is reported in Figure 3.

### 3.3. Correlation between the Severity Score and Total Lymphocytes

**Figure 4:**
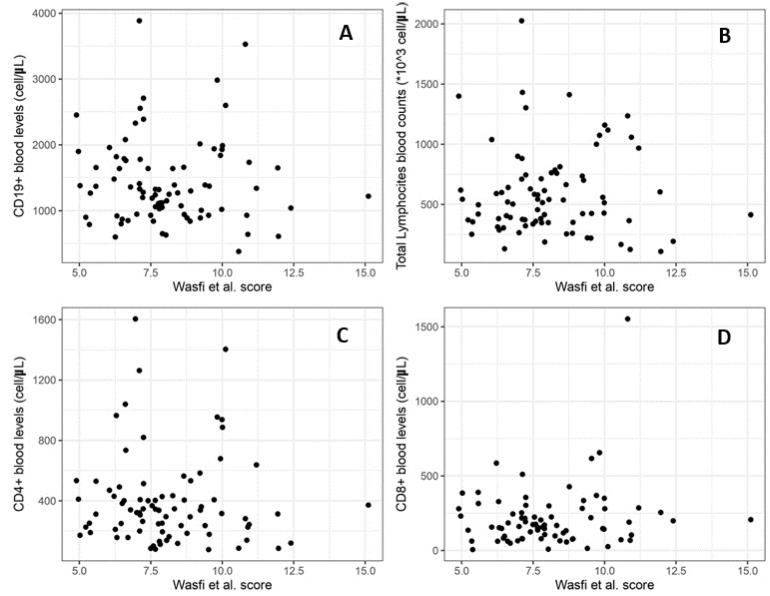
Correlation between Wasfi et al. [24] severity index score (x-axis of graph) and total lymphocyte count (**A**), CD4+ blood levels (**B**), CD8+ (**C**), and CD19+ (**D**).

We then calculated the correlation between the Wasfi et al. [24] score and blood levels of total lymphocytes and then with the subpopulation of CD4+, CD8+, and CD19+, as shown in Figure 4. None of the associations resulted statistically significant, not even at a suggestive level. The correlation between the severity score and total lymphocytes shows a r = −0.086 (*p*-value = 0.44). We then calculated the correlation with the level of CD4+ which results in r = 0.028 (*p*-value = 0.80), CD8+ with r = −0.113 (*p*-value = 0.31), and CD19+ with r = 0.045 (*p*-value = 0.44).

**Figure 5:**
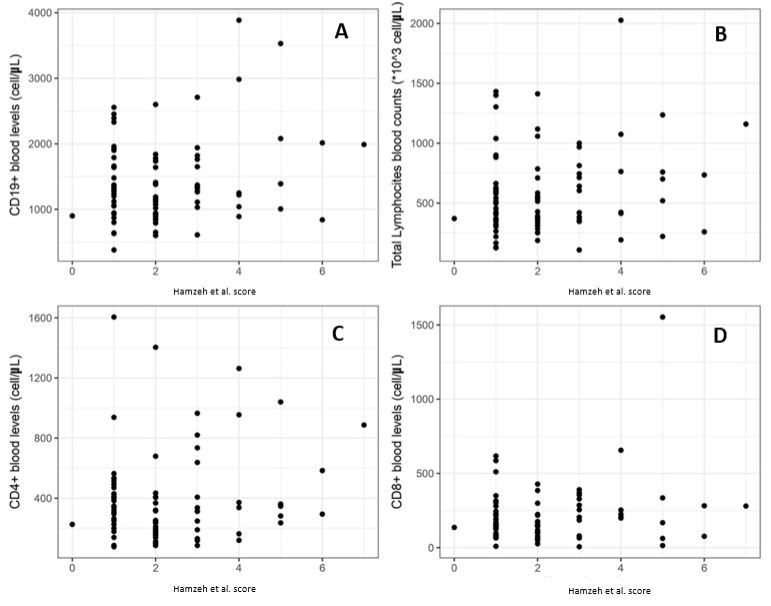
Correlation between Hamzeh et al. [25] severity index score (x-axis of graph) and total lymphocyte count (**A**), CD4+ blood levels (**B**), CD8+ (**C**), and CD19+ (**D**).

Finally, we analyzed the correlation between the Hamzeh et al. [25] severity score and the previous parameters, as shown in Figure 5. Also, in this case, no significant associations were detected between the severity score considered and the level of lymphocytes in peripheral blood. The total lymphocytes demonstrate a r = 0.122 (*p*-value = 0.28), CD4+ a r = 0.148 (*p*-value = 0.19), CD8+ a r = 0.034 (*p*-value = 0.77) and CD9+ a r = 0.120 (*p*-value = 0.30). 

## 4. Discussion

In this study, we aimed to evaluate in sarcoidosis patients whether immune system alterations detected by lymphocyte typing in peripheral blood can be correlated with the severity of the disease and thus guide physicians in their approach to these patients. However, in our population study, lymphocyte subpopulation values at the time of diagnosis do not appear to correlate with sarcoidosis severity at onset. In recent years, numerous studies have shown that sarcoidosis is a very complex disease, both symptomatically and in terms of its pathophysiology. 

The cause of sarcoidosis is unknown [2,3,4,5,6]. Several studies reported that this disease may be due to an immune reaction to a trigger such as an infection or chemicals in those who are genetically predisposed [5,6,7,8,9,10]. Those with affected family members are at greater risk [4]. Diagnosis is partly based on signs and symptoms, which may be supported by biopsy [6]. Furthermore, the diagnosis should only be made after excluding other possible causes of similar symptoms, such as tuberculosis [6,17].

The latest research has moved by trying to identify the immunological reason underlying the development of this disease. Reduced T-lymphocyte activity is associated with reduced INFγ production, the involvement of underactive CD17+ and iNKTs, and poor cytokine production. Too little active B cells probably underlie the development of the disease [9,11,12,13]. However, there are no biomarkers that can predict whether, at the time of diagnosis, the disease will resolve spontaneously or not. In fact, the disease may resolve without any treatment within a few years [2,3,4,5]. However, some patients may have long-term or severe disease [2,3,4,5,6,7]. In particular, some symptoms may be improved with the use of anti-inflammatory drugs such as ibuprofen [26,27]. In cases of important symptoms and signs, steroids such as prednisone are indicated [27,28]. Conventional disease-modifying antirheumatic drugs (cDMARDs), such as methotrexate, chloroquine, or azathioprine, and biological disease-modifying antirheumatic drugs (bDMARDs), such as infliximab and adalimumab, can be used in cases where steroids are not sufficient to control the disease and in an attempt to reduce the side effects of steroids [9]. The risk of death is 1–7% [5]. The chance of the disease returning in someone who has had it previously is less than 5% [2].

This study, therefore, investigates whether the level of lymphocytes in peripheral blood can be used as a marker of chronic disease, answering the question: is lymphopenia in peripheral blood related to chronic sarcoidosis? However, our results show that none of the two scores (that moderately correlated to each other) show an association with the level of total lymphocytes or with the DC4+, DC8+, or DC19+ lymphocyte subclasses. Thus, the level of lymphocytes in peripheral blood is not a good marker of the severity of sarcoidosis. A limitation of this study was the assessment of lymphocyte levels only at onset and not during the disease, although therapy may influence the level. In addition, the scores used have limitations of incompleteness; both scores do not assess disease activity as a signal of increased activity by PET or gallium scintigraphy and do not consider the amount and type of therapy required to control the disease. Furthermore, another limitation of our work is the sample size of our study population.

Going deeper into the rationale behind the development of the two scores, it is good to specify that Wasfi et al. considered 104 patients with a diagnosis of sarcoidosis confirmed by histological examination, according to the guidelines of the American Thoracic Society consensus panel of the time [24]. The involvement of various organs and systems was based on the Case Control Etiologic Study of Sarcoidosis (ACCESS) assessment instrument [29]. Radiological involvement was defined by analyzing a chest X-ray taken within one year of study involvement and examined by two different pulmonologists experienced in staging sarcoidosis according to the radiological score proposed by Scadding [30]. Furthermore, the patient’s ethnicity was taken into account, as it is known from several previous studies that the Afro-American race has a higher rate of extrathoracic involvement with poor prognosis [30,31]. Wasfi et al. also proposed an equivalent risk score formula that does not, however, consider spirometric data, as these are not always available. We did not take this alternative formula into account in our study, as all the patients examined had valid respiratory function data available at the time of diagnosis [24]. 

Regarding the second severity score considered, Hamzeh et al. [25] tried to propose a scoring system that could go beyond the Scadding score, as it takes into account both the radiological involvement of sarcoidosis according to an already known pattern and the lung function at the time of diagnosis. Hamzeh’s score was then further validated by subjecting 92 patients with sarcoidosis to cardiopulmonary exercise testing (CPET) and finding a statistically significant correlation between CPET ventilatory and gas exchange measurements and the purposed score [25]. The main reason for the development of this score was, therefore, to propose a classification that would better express the respiratory function changes that characterize sarcoidosis than the Scadding Score, proposed and validated in the 1960s [32]. Several studies have, in fact, already highlighted the need to evolve and go beyond the Scadding score, proposing different classifications of sarcoidosis capable of better phenotyping this disease [31,32]. 

The first study in the literature to detect alterations in lymphocyte subpopulations in patients with sarcoidosis was conducted in 2010 by Sweiss et al. in 28 patients with sarcoidosis. Unlike our results, a correlation was found between disease severity, in terms of multiorgan involvement, and lymphocyte subpopulation levels. It should be noted, however, that, unlike our work, Nadera et al. did not use severity scores to uniquely assess the severity of sarcoidosis. Indeed, in this study, the severity of the disease was determined according to the number and extent of involvement of various organs and apparatuses, particularly neurological, cardiac, ocular, and advanced pulmonary involvement. In contrast to our study, Sweiss et al. did not consider patients according to Scadding’s classification, and the use of Hamzeh’s score in our study allowed us to assess patients not only according to organ involvement but also on the basis of lung function at the time of diagnosis [32,33]. However, one aspect observed by Sweiss et al. that was not taken into account in our study was the absence of a difference in lymphocyte subpopulations in patients taking different classes of drugs for the treatment of sarcoidosis (such as methotrexate, azathioprine, leflunomide, mycophenolate mofetil, prednisone, or tumor necrosis factor-alpha inhibitors). 

## 5. Conclusions

To our knowledge, this is the first study conducted in a cohort of sarcoidosis patients, which tested whether immune system alterations detected by lymphocyte typing in peripheral blood correlated with the severity of disease, calculated according to two separate severity scores proposed by Wasfi in 2006 and Hamzeh in 2010 [24,25].

Our findings suggest no correlation between the blood levels of total lymphocytes and their subpopulations at the time of diagnosis and disease severity. We can conclude that the levels of total lymphocytes, CD4+ T lymphocytes, CD8+ T lymphocytes, and CD19+ T lymphocytes cannot currently be used as a marker of disease severity at diagnosis. However, additional research and analysis should be carried out before making any concrete conclusions. At this point, the prospects of future studies may go in two directions: First, the creation of a new, more comprehensive severity score. Second, further deepening the knowledge of immune system involvement in all stages of the disease and the search for specific biomarkers that are already at the onset of sarcoidosis can help us predict the course of the disease and, thus, the need for appropriate treatment. Furthermore, new studies are ongoing to evaluate the possible correlation between the course of the disease and the changes in the risk scores for extending the knowledge on this rare disease.

## Figures and Tables

**Table 1:** Sarcoidosis’ manifestation, nonspecific or specific, as considered in our study.

Organ Involvement	Symptoms
Nonspecific	Fatigue and weakness, fever, weight loss, night sweats, and malaise
Pulmonary	Nonproductive cough, dyspnea on exertion or at rest, expectoration, bilateral hilar adenopathy
Skin	Erythema nodosum, Lupus pernio, other injuries consistent with sarcoidosis at histological exam
Central nervous system	Cranial neuropathies, myelopathy, polyneuropathy, mononeuritis multiplex, small-fiber neuropathy
Cardiac	Arrhythmias, cardiomyopathy, sudden death
Musculoskeletal and Joint	Polyarthritis, bone cysts, Achilles tendonitis, dactylitis, heel pain, myopathy
Ocular	Anterior and posterior uveitis, chorioretinitis, conjunctivitis, optic neuritis, glaucoma
Renal	Renal calculi, renal failure, epididymitis
Hepatic	Hepatomegaly, cirrhosis

**Table 2:** Patients’ characteristics considered in our study.

Patients’ Characteristics	
*Gender, n (%)*	
Male	46 (57%)
Female	35 (57%)
*Age, mean (SD)*	57 (12%)
*Former smokers, n (%)*	34 (42%)
*Onset symptoms n, (%)*	
Pulmonary	74 (91%)
Skin	23 (28%)
Joint and muscles	20 (25%)
Cardiac	16 (20%)
*Former smokers, n (%)*	34 (42%)
*Organ involvement n, (%)*	
Lymph node involvement	59 (73%)
Lung	71 (88%)
Renal/Hypercalciuria	24 (29%)
Cutaneous	23 (28%)
Osteoarticular	20 (25%)
Abdominal	10 (12%)
Cardiac	16 (20%)
Neurological	6 (7%)
Ocular	16 (20%)
Lofgren’s syndrome	11 (14%)
*Scadding score n, (%)*	
Stage 0	2 (2.5%)
Stage I	8 (9.9%)
Stage II	39 (48%)
Stage III	15 (19%)
Stage IV	17 (21%)
*FEV1 n, (%)*	
70–100%	68 (84%)
50–69%	11 (14%)
<50%	2 (2.5%)
GCs* therapy n, (%)	79 (98%)
Non GCs* therapy n, (%)	58 (72%)
*High ACE levels n, (%)*	26 (32%)
*Wasfi score, median (IQR)*	7.78 (6.79, 9.27)
*Hamzeh score, median (IQR)*	2 (1, 3)

GCs*: Glucocorticoids.

**Table 3:** Hamzeh et al. [25] Sarcoidosis severity score.

Sarcoidosis Characteristics	Points
Scadding stage	
Stage 0 and I	0 pts.
Stage II and III	1 pts.
Stage IV	2 pts.
Forced Vital Capacity (FVC, %)	
>80%	0 pts.
60–79%	1 pts.
<59%	2 pts.
Tiffeneau Index	
>70%	0 pts.
≤70%	1 pts.
DLCO (%)	
>80%	0 pts.
60–79%	1 pts.
<59%	2 pts.

**Table 4:** Flow cytometry results.

Measure	Median	Q1	Q3
Total lymphocyte	1300	947	1763
CD4+	515	355	735
CD8+	322	200	434
CD19+	168	96	280

## Data Availability

All the data are available upon reasonable request to the corresponding author.
